# Gas-phase volatilomic approaches for quality control of brewing hops based on simultaneous GC-MS-IMS and machine learning

**DOI:** 10.1007/s00216-020-02842-y

**Published:** 2020-08-04

**Authors:** Rebecca Brendel, Sebastian Schwolow, Sascha Rohn, Philipp Weller

**Affiliations:** 1 Institute for Instrumental Analytics and Bioanalytics, Mannheim University of Applied Sciences, Paul-Wittsack-Strasse 10, 68163 Mannheim, Germany; 2 Institute of Food Chemistry, Hamburg School of Food Science, University of Hamburg, Grindelallee 117, 20146 Hamburg, Germany

**Keywords:** Simultaneous HS-GC-MS-IMS, Machine learning, Volatilomics, Brewing hops, Quality control, Partial least square regression (PLSR)

## Abstract

For the first time, a prototype HS-GC-MS-IMS dual-detection system is presented for the analysis of volatile organic compounds (VOCs) in fields of quality control of brewing hop. With a soft ionization and drift time-based ion separation in IMS and a hard ionization and *m/z*-based separation in MS, substance identification in the case of co-elution was improved, substantially. Machine learning tools were used for a non-targeted screening of the complex VOC profiles of 65 different hop samples for similarity search by principal component analysis (PCA) followed by hierarchical cluster analysis (HCA). Partial least square regression (PLSR) was applied to investigate the observed correlation between the volatile profile and the α-acid content of hops and resulted in a standard error of prediction of only 1.04% α-acid. This promising volatilomic approach shows clearly the potential of HS-GC-MS-IMS in combination with machine learning for the enhancement of future quality assurance of hops.

**Electronic supplementary material:**

The online version of this article (10.1007/s00216-020-02842-y) contains supplementary material, which is available to authorized users.

**Figure:**
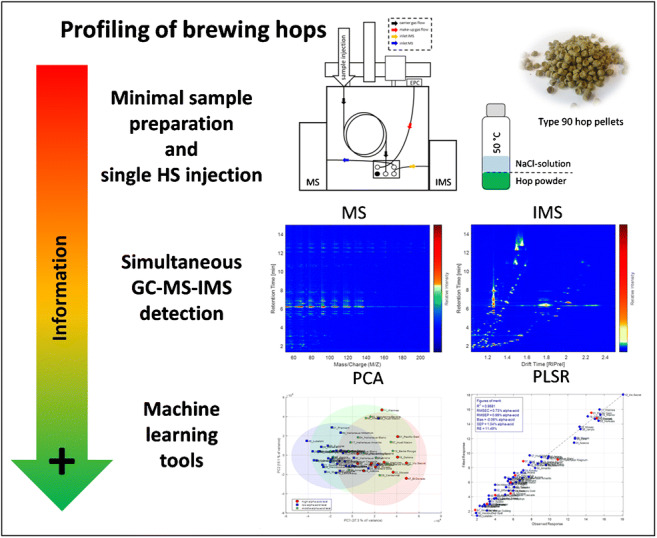
Graphical abstract

## Introduction

For centuries, hop (*Humulus lupulus* L.) has been used in the brewing process, due to its contents of secondary metabolites that give beer its typical flavor. Among those, the α- and β-acids (often referred to as *bitter acids*) formed in the lupulin glands of the hop cone are mainly responsible for bitter taste of beer [1]. In contrast, terpenes and terpenoids of the essential oil fraction and their oxygenated derivatives are responsible for the hoppy and spicy beer flavor [2]. Even today, the hop price and also the hop dosage mainly depend on the bitter acid level of the hop blossoms and, in particular, the α-acid content that mostly contribute to the bitterness during wort cooking [1]. With the increasing craft beer movement, special flavor hops currently gain great popularity. For those hop cultivars, the aroma yield or aroma potential is even more meaningful in terms of suitability and authenticity [3]. The bitter acid content as well as the essential oil composition is influenced by several factors, among of which the date of harvesting is of particular importance. α-Acid and essential oil contents increase significantly within the last few days prior to harvesting and are highly variable among cultivars [4]. Furthermore, cultivation and processing conditions play an important role. It was shown that hop quality is significantly influenced by climate change effects, such as extremely hot and dry summers, which led to poor yields and low α-acid levels [1, 5, 6]. Additionally, pathogens such as the citrus bark cracking viroid (CBCDv) may drive losses in crop yields [1, 5, 7]. The evaluation of hop quality bases typically on the chemical analysis of the essential oil and bitter acid contents, next to sensory evaluation and visual inspection. The *European Brewery Convention* (EBC) and the *American Society of Brewing Chemists* (ASBC) established a collection of validated analytical methods for the routine analysis of hop. For the unspecific determination of the α-acid content, the EBC 7.4 method can be applied, including the conductometric determination of the lead conductance value by the reaction of lead acetate and α-acids, as well as the ASBC method for the photometric determination of the bitter acids [1]. For the specific determination of the bitter acids, the EBC 7.7 method, using high-performance liquid chromatography (HPLC) followed by diode array detection or mass spectrometry, is usually utilized [1]. Both methods require liquid extraction of the soft resin in hops with methanol and diethyl ether prior to analysis. The determination of the essential oils bases on a time-consuming steam distillation according to EBC 7.10, followed by GC-MS or GC-FID analysis [1]. As a result, a number of methods have been developed to reduce the experimental effort required. Field et al. used a direct headspace solid-phase microextraction (SPME) coupled to GC-FID instead of steam distillation for the quantification of specific terpenes [8], while Aberl and Coelhan described a headspace-trap GC-MS approach [9]. Čulík et al. used accelerated solvent extraction to speed up the solvent extraction procedure for bitter acids [10]. Furthermore, the *hop storage index* (HSI) based on UV/Vis spectroscopy was established according to EBC 7.13 as a reference value for hop aging. Storage tests demonstrated that the bitter acid content decreases steadily and the composition of the essential oils changes due to oxidation reactions, but the deterioration process can be reduced significantly by inert storage conditions at low temperatures [11–13]. As HSI, the ratio of the absorption at 275 nm, which is characteristic for the oxidation products of bitter acids, and at 325 nm, which is characteristic for the fresh bitter acids, is calculated [1, 12]. However, this method does not deliver detailed information about the hop quality itself. An indicator for flavor compound aging is the epoxide fraction, and therefore, epoxides of caryophyllene and humulene are analyzed in relation to the non-epoxidized forms. This procedure again is time-consuming due to the required extraction procedure and GC analysis.

Multivariate data analysis has already been applied in the quality control of hop, mostly for authenticity control [14, 15]. Ocvirk et al. used multivariate cluster analysis (CA) for the varietal classification of five different hop varieties concerning the bitter acid and essential oil composition [14] that are known to be characteristic for each hop cultivar [12, 13, 16]. Kovačevič et al. also used GC-FID combined with principal component analysis (PCA) and CA for the verification of hop varieties [15]. Farag et al. used metabolomic fingerprints obtained by ^1^H-nuclear magnetic resonance (NMR) and Fourier transform ion cyclotron resonance (FT-ICR)-MS for cultivar classification [17], as well as the signal pattern derived from two-dimensional NMR [18]. Killeen et al. applied partial least square regression (PLSR) for the quantification of bitter acids by vibrational spectroscopy [19] and could distinguish different hop cultivars due to their bitter acid composition by applying principal component analysis to data derived from Raman and ^1^H-NMR spectroscopy [16]. Nonetheless, there is one major disadvantage of NMR and FT-ICR-MS: The complex instrumentation technique requires much maintenance and technical support next to high purchasing costs.

In the last decade, ion mobility spectrometry (IMS) with radioactive ionization in combination with chemometrics gained popularity in food analysis [20–23]. Gerhardt et al. used headspace GC-IMS (HS-GC-IMS) for the classification of virgin olive oils [20, 22] as well as for authenticity control of honey [21]. In drift time IMS, ions are separated in the drift tube at atmospheric pressure, according to their mass, charge, and collision cross section (CCS), resulting in a high resolving power. Proton affine substances such as esters, aldehydes, and ketones are reported to be detectable in trace levels, because they are mainly ionized by proton transfer reactions [24]. Furthermore, the use of low radiation tritium ion sources of only 300 MBq requires no license accordance to EU directive 29/96 EURATOM [25]. IMS works as highly selective, sensitive, and very robust detection system without requiring high technical effort. In combination with chemometrics, HS-GC-IMS represents an excellent tool for flavor profiling without requiring time-consuming sample preparation procedures. Specifically, the 2D data generated by GC-IMS are highly suitable for complex compound fingerprints as being found in hops, which was shown in a feasibility study limited to German hop samples by Kurzweil et al. [26]. Optimally, these 2D data and data from MS techniques could be combined to a more complete view on the flavor compound fingerprint.

Consequently, the aim of this study was the development of a fast and cost-efficient benchtop *volatilomics* strategy for the flavor profiling of hops to evaluate quality and to lay the basis for authenticity control. The focus was set on minimal sample preparation, robustness, and ease of use. For this purpose, a HS-GC-MS-IMS prototype is presented for the first time. This setup allows the parallel detection of MS and IMS spectra by single injection resulting in an increased acquisition of information and the opportunity of the direct comparison of both techniques for substance identification. A further aim was the development of a suitable data analysis and machine learning toolset for the highly complex data generated by the prototype setup to classify hop cultivars and potentially, to quantitate the α-acid content via the gas phase. To the best of our knowledge, simultaneous GC-IMS-MS has not been used for the analysis of hops so far.

## Material and methods

### Reagents and sample preparation

Type 90 hop pellets were used with regard to a higher homogeneity of the hop samples. One gram of type 90 pellets of 65 different commercially available brewing hops (listed in Electronic Supplementary Material (ESM) in Table S1) was dried with liquid nitrogen and ground by mortar and pestle. A total of 100 ± 10 mg of the resulting hop powder was transferred to a 20-mL headspace vial, 2 mL of a sodium chloride solution (300 g/L deionized water) was added, and the vial was sealed with a screw cap. All samples were measured in duplicate. Prior to sample preparation, the hops were stored in vacuum seal bags at 4 °C protected from light.

Stock solutions of reference compounds were prepared in sunflower oil (GLOBUS-Holding GmbH & Co. KG, St. Wendel, Germany) as solvent and in the concentration range of 10 mg/g for myrcene (Sigma Aldrich GmbH, Steinheim, Germany), *β-*caryophyllene (Carl Roth GmbH & Co. KG, Karlsruhe, Germany), and *α-*humulene (Sigma Aldrich); 1 mg/g for linalool (Sigma Aldrich) and limonene (Sigma Aldrich), as well as 0.1 mg/g for *α-*pinene (Acros Organics™ by Thermo Fisher GmbH, Kandel, Germany), and *β-*pinene (Alfa Aeser by Thermo Fisher GmbH, Kandel, Germany).

### Instrumentation

All sample measurements were performed on a prototype, dual-detector HS-GC-MS-IMS system, based on an Agilent 5973 mass spectrometer (Agilent Technologies Deutschland GmbH, Waldbronn, Germany) and an OEM ion mobility spectrometer module (Gesellschaft für Analytische Sensorsysteme mbH, Dortmund, Germany), coupled to an Agilent 6890 GC (Agilent Technologies) via a three-way Dean’s switch plate (Agilent Technologies), to compensate for the pressure differences in the detection systems (ESM Fig. S1). Detection was carried out in parallel on both detectors. HS sample injection was performed using a CombiPAL autosampler (CTC Analytics AG, Zwingen Switzerland), equipped with a static headspace sampling unit and a gas-tight 2.5-mL heatable syringe (Hamiltion, Reno, USA) at 60 °C to avoid condensation effects. A headspace volume of 1 mL was injected into a split/splitless injector operated at 250 °C and a split ratio of 1:10, after an incubation time of 10 min at 50 °C and 500 rpm. The syringe was flushed for 3 min before each sample injection cycle to avoid carry over. A ZB-5ms column (30 m × 0.25 mm × 0.25 μm, Phenomenex Inc., Torrance, USA) was installed in the GC operated with a carrier gas flow of 4 mL/min of helium and an oven program of 40 °C initial temperature followed by a temperature ramp of 10 °C/min to 250 °C hold for 5 min (total run time: 26 min). After chromatographic separation, the gas flow was split at the splitter plate and transferred to two identical retention gaps of 1-m length and 0.1-mm inner diameter, each leading to one of the two coupled detectors. An additional make-up gas flow of helium was added by a custom EPC regulator at the split point for maintaining a sufficient gas flow of 2.3 mL/min to IMS in order to reduce peak broadening. Heated transfer lines were operated at 200 °C (IMS) and 250 °C (MS), respectively.

The IMS module was equipped with a ^3^H radioactive ionization source. A voltage of 5 kV was applied to the drift tube of 9.8-cm length operated at 90 °C with a constant nitrogen drift gas flow of 150 mL/min of 99.9999% gas purity. A number of six IMS spectra were averaged, respectively, each recorded in positive mode with a repetition rate of 21 ms, an injection pulse width of 150 μs, and a blocking and injection voltage of 70 and 2500 mV, respectively.

The MS consisted of an electron impact (EI) ion source operated with an ionization energy of 70 eV at 230 °C and a single quadrupole at 150 °C scanning a mass range of *m/z* 50–550.

### Data pre-processing

All obtained spectra were concatenated to an array (number of samples × retention times × drift times or mass to charge (*m/z*) ratios) for IMS and MS data, respectively. Savitzky-Golay smoothing was applied to both data sets to reduce random noise. Drift time (DT) and retention time (RT) alignment was applied to the IMS data followed by interpolation of the DT axis. The DT was normalized to the reactant ion peak (RIP) position and the RT to the RT of nitrous oxide visible in each spectrum. MS data were aligned in RT direction only and log transformation was applied. Afterwards, the amount of data was reduced by extracting the region of relevant signals between 1.3 and 15 min in each spectrum. Finally, replicated measurements were summarized to one single mean spectrum per sample resulting in two data sets of a 65 × 2785 × 1554 (sample number × RT × DT) cube of IMS data and a 65 × 2392 × 160 (sample number × RT × *m/z* ratios) cube of MS data. Before applying the different algorithms, the three-dimensional arrays were unfolded to two-dimensional matrices, respectively, and mean centering was applied.

### Chemometric data analysis

For reduction of dimensionality and for data visualization, principal component analysis (PCA) was applied to both data sets as unsupervised multivariate analysis method. For the comparison of the aroma profiles of the different cultivars, hierarchical cluster analysis (HCA) was applied including the first five principal components (PCs) obtained by PCA. The distance between clusters was determined by Ward’s method with Euclidean distances as distance metric. Based on the results of the PCA score plot, partial least square regression (PLSR) was used to investigate the potential correlation. The PLSR model was built on a training set of 55 samples and a response vector containing the corresponding α-acid concentration values. The test set of the remaining samples was used for model validation covering the whole calibration range of α-acid concentrations. Figures of merit were calculated as shown in the section below. To prevent overfitting, the optimum number of latent variables (LVs) was determined by considering the validation results by stepwise increase of the number of LVs. Finally, PLSR loading plots were used for the identification of volatile organic compounds (VOCs) responsible for the observed correlation.

### Figures of merit

To determine the quality of the applied PLSR model, the determination coefficient (*R*^2^) and the root mean square error of calibration (RMSEC) were calculated:

1 $$ {R}^2=1-\frac{\sum_{i=1}^n{\left({c}_i-{c}_i^{\mathrm{reg}}\right)}^2}{\sum_{i=1}^n{\left({c}_i-\overline{c}\right)}^2} $$

2 $$ \mathrm{RMSEC}=\sqrt{\frac{\sum_{i=1}^n{\left({c}_i-{c}_i^{\mathrm{reg}}\right)}^2}{n}} $$

where *c*_*i*_ is the actual concentration of the training set, ${c}_i^{\mathrm{reg}}$ the corresponding concentration calculated by the built model, $\overline{c}$ the arithmetic mean of *c*_*i*_, and *n* the number of samples of the training set.

For validation the root mean square error of prediction (RMSEP)

4 $$ \mathrm{RMSEP}=\sqrt{\frac{\sum_{i=1}^n{\left({c}_i-{c}_i^{\mathrm{pred}}\right)}^2}{n}}, $$

the systematic error (Bias)

5 $$ \mathrm{Bias}=\frac{\sum_{i=1}^n\left({c}_i-{c}_i^{\mathrm{pred}}\right)}{n}, $$

the standard error of prediction (SEP)

6 $$ \mathrm{SEP}=\sqrt{\frac{\sum_{i=1}^n{\left({c}_i-{c}_i^{\mathrm{pred}}-\mathrm{Bias}\right)}^2}{n-1},} $$

and the relative percentage error of prediction (RE) were calculated

7 $$ \mathrm{RE}\ \left(\%\right)=100\sqrt{\frac{\sum_{i=1}^n{\left({c}_i-{c}_i^{\mathrm{pred}}\right)}^2}{\sum_{i=1}^n{c_i}^2},} $$

where *c*_*i*_ is the actual concentration of the test set, ${c}_i^{\mathrm{pred}}$ the corresponding predicted concentration calculated by the regression model, and *n* the number of samples of the test set.

### Software

For mass spectral data acquisition and mass spectra comparison, the MSD ChemStation Software (Agilent Technologies) was used. Data export and Savitzky-Golay smoothing of the IMS data were performed with the LAV Software version 2.2.1 (Gesellschaft für Analytische Sensorsysteme mbH). Further data pre-processing of IMS and MS data, model building, validation, and calculation of figures of merit were implemented in own MATLAB routines and carried out in MATLAB (The MathWorks Inc., Natick, MA, USA) using the Statistics and Machine Learning Toolbox (MathWorks).

## Results and discussion

A number of 65 different pelletized samples of hop cultivars from harvesting years 2015–2018 (ESM Table S1) were analyzed by a prototype HS-GC-MS-IMS setup. Hop pellets are mixtures of different hop lots for balancing the variability caused by growing conditions, storage, and other effects. Further, such a mixing enables better storage stability [27, 28].

### Direct comparison of IMS and MS data

**Fig. 1:**
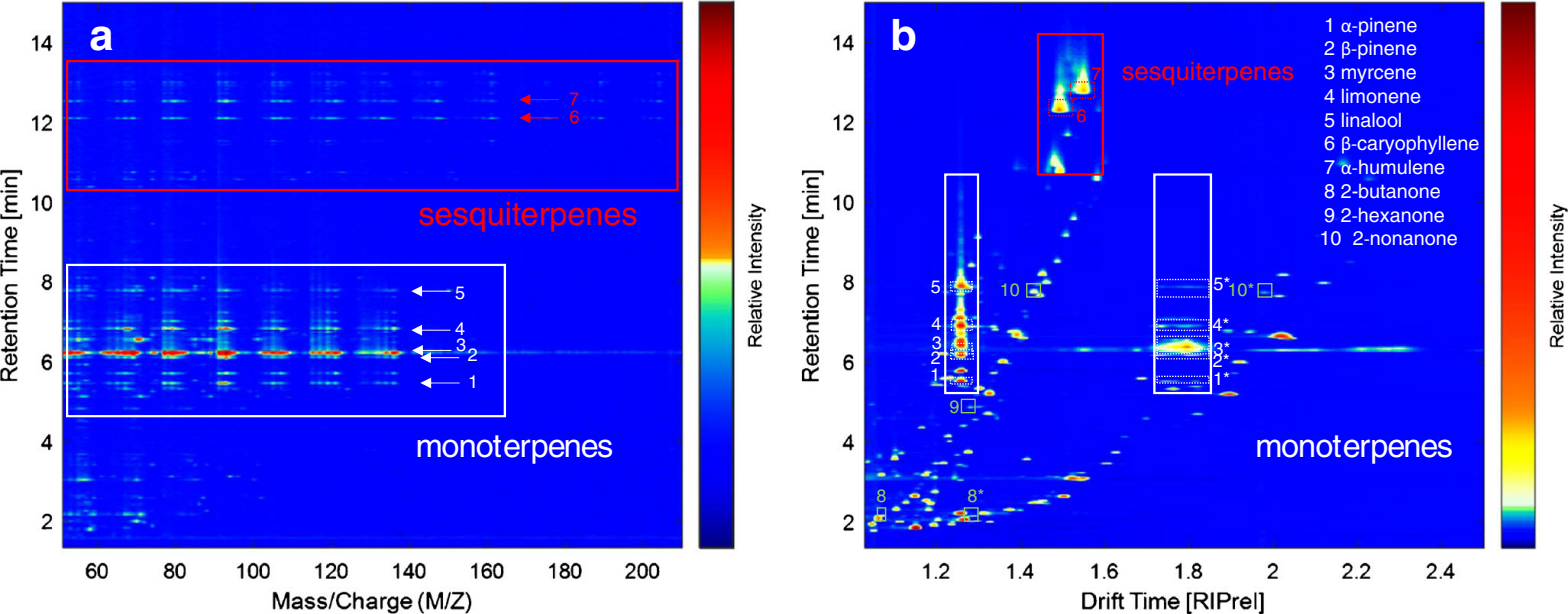
2D spectra of the hop variety Citra measured by EI-MS (**a**) and IMS (**b**). The regions for monoterpene ion signals are highlighted in white, for sesquiterpene ion signals in red, respectively. All identified substances are labelled with arrows in the MS spectrum or surrounded by dashed lines in the IMS spectrum and belong to α-pinene (1), β-pinene (2), myrcene (3), limonene (4), linalool (5), β-caryophyllene (6), α-humolene (7), 2-butanone (8), 2-hexanone (9), and 2-nonanone (10). Higher molecular cluster ions (dimers) are indicated by an asterisk

IMS and MS spectra were recorded in parallel from each sample by the HS-GC-MS-IMS setup. The exemplary volatile profiles of the hop variety Citra are shown in Fig. 1. For a better direct comparison, the MS data are given in form of a 3D heatmap (retention time × *m*/*z* × intensity), while IMS data are given as retention time × drift time × intensity. Due to the significantly high abundance of myrcene in hops, the MS data were log-transformed in order to allow a graphical comparison as given in Fig. 1.

It can be seen that IMS spectral data are much denser and complex compared with the EI-MS data. The reason for this effect may be explained by two aspects: On the one hand, terpenes show a strong fragmentation behavior under EI conditions, which results in very similar fragments for different isomers or closely related compounds. This leads to the similar patterns observed in the monoterpene and sesquiterpene zone. On the other hand, IMS provides a clearly softer ionization, which leads to more stable ions being additionally separated by their ion-neutral CCS values. However, both techniques have their distinct characteristics: Mono- and sesquiterpenes, the dominating species of the hop essential oil, can be distinguished in EI-MS (Fig. 1a) by their fragmentation behavior, resulting in dominating fragments of *m*/*z* 136, 121, 93, and 69 for monoterpenes (white box) and *m*/*z* 204, 121, 93, and 69 for sesquiterpenes (red box). In general, unequivocal differentiation of isomeric terpenes in unit-resolved MS is only feasible when comparing the spectra of reference compounds. The fragmentation behavior is highly structure-dependent with regard to differences in fragment ion abundances. Consequently, in the case of co-elution of substances of similar fragmentation behavior, this information is interfered. In IMS spectra, monoterpenes and sesquiterpenes can be distinguished better due to the 2D separation with different normalized drift times of 1.258 (monomers) and 1.711–1.858 (dimers) for monoterpenes, or respectively 1.477 and 1.567 (monomers) for the sesquiterpenes (Fig. 1b). Isomeric terpenes often feature monomers of identical mobility, but with different relative abundances of their dimers or a variation in the number of product ions. Interestingly, terpene alcohols such as linalool show ion patterns with similar drift times as the corresponding terpenes, although the molecules differ in mass and CCS values. This behavior has already been reported for isomeric hydrocarbons or hydrocarbons with different substituents by Borsdorf and Neitsch [29], indicating a complex underlying ion chemistry during the ion formation process, such as elimination or rearrangement reactions. From the IMS spectra, it is clearly visible that there are co-eluting compounds in the monoterpene region of the IMS spectrum (Fig. 1b). Here, the complementary separation of GC and IMS allows distinguishing these compounds from the terpene signals, because their DT clearly differs. These signals belong to minor volatile components of hops, such as ketones, aldehydes, carboxylic acids, and esters of different chain length and branch type [1, 2, 9, 13], which are reported to be byproducts from terpene biosynthesis and from degradation processes of lipids and bitter acids [2]. An exemplary set of substances was identified as 2-butanone (8), 2-heptanone (9), and 2-nonanone (10) via reference standards in the GC-IMS data. The EI-MS spectrum features a much more complex fragmentation pattern. Here, a time-consuming comparison of fragments and their abundances versus reference substance spectra is required for the identification without further information about the identity of the interfering substance. In contrast, the IMS spectra allow identifying substances more easily or at least assign these substances to a substance class; however, the combination of MS and IMS data may lead to an even more simplified identification due to the complementary information.

At the current stage, extensive libraries of IMS drift time spectra are not yet available. For this reason, an in-house generated reference library of hop aroma compounds was built for similarity-based search in obtained mass and drift time spectra. For MS data, the probability-based matching algorithm (PBM), which uses *m*/*z* as well as abundance values, was applied for mass spectral comparison implemented in the ChemStation Software [30] and resulted in the match quality value representing the degree of similarity between the spectra. The calculation of a match quality value for IMS spectra is less straightforward, because the number of ions and relative abundance is highly dependent on concentration. For this reason, RT and DT were only used for substance identification. Commonly known, simple mono- and sesquiterpenes as well as monoterpene alcohols of the hop essential oil fraction were identified. RT, match quality values, RIP-normalized DT, and *K*_0_ values are listed in Table 1.

**Table 1:** Results of the substance identification of common terpenes and terpene alcohols of the hop variety Citra

Number	Name (molecular mass)	EI-MS		IMS	
		Retention time^MS^ (min)	Match quality value^MS^	Retention time^IMS^ (min)	Drift times (RIPrel)* (*K*_0_ [cm^2^/Vs])**
1	α-Pinene (136.23 g/mol)	5.47	90	5.52	1.258, 1.745, 1.767, 1.818 (1.630, 1.175, 1.160, 1.128)
2	β-Pinene (136.23 g/mol)	6.11	93	6.17	1.258, 1.735, 1.808 (1.630, 1.182, 1.134)
3	β-Myrcene (136.23 g/mol)	6.23	91	6.30	1.258, 1.769, 1.795 (1.630, 1.159, 1.142)
4	Limonene (136.23 g/mol)	6.82	38	6.88	1.258, 1.736, 1.795 (1.630, 1.181, 1.142)
5	Linalool (154.25 g/mol)	7.78	46	7.87	1.258, 1.736, 1.787 (1.630, 1.181, 1.147)
6	β-Caryophyllene (204.36 g/mol)	12.11	96	12.32	1.494, 1.586 (1.372, 1.293)
7	α-Humulene (204.36 g/mol)	12.56	90	12.82	1.552 (1.321)

*The drift times are normalized to the reactant ion drift time

**K_0_ $=K\left(\frac{273}{T}\right)\left(\frac{P}{760}\right)$ with $K=\frac{L}{t_{\mathrm{d}}E}$, where *L* is the drift tube length (cm), *t*_d_ is the drift time (s), *E* is the electrical field (V/cm), *T* is the drift tube temperature (K), and *P* is the pressure inside the drift tube (Torr)

The comparison of the RTs from both techniques shows only slight deviations of 0.05–0.09 min, while for the sesquiterpenes, deviations between 0.21 and 0.26 min were observed. This slight increase in RT for later eluting compounds can be attributed to peak broadening, caused by adsorption effects in the non-heated ionization cell of the IMS, being already considerably reduced by an additional make-up gas flow at the Dean’s switch plate after the analytical column. This approach delivered nearly identical chromatographic conditions for both detection systems, which is, to our knowledge, described for the first time for the combination of IMS and MS detection in GC. Budzyńska et al., who used a parallel injection with two independent injection lines for IMS and MS [31], were not able to directly correlate the resulting data, because of strongly differing retention behavior. Furthermore, it was virtually impossible to ensure identical chromatographic conditions, being crucial for the subsequent data analysis.

**Fig. 2:**
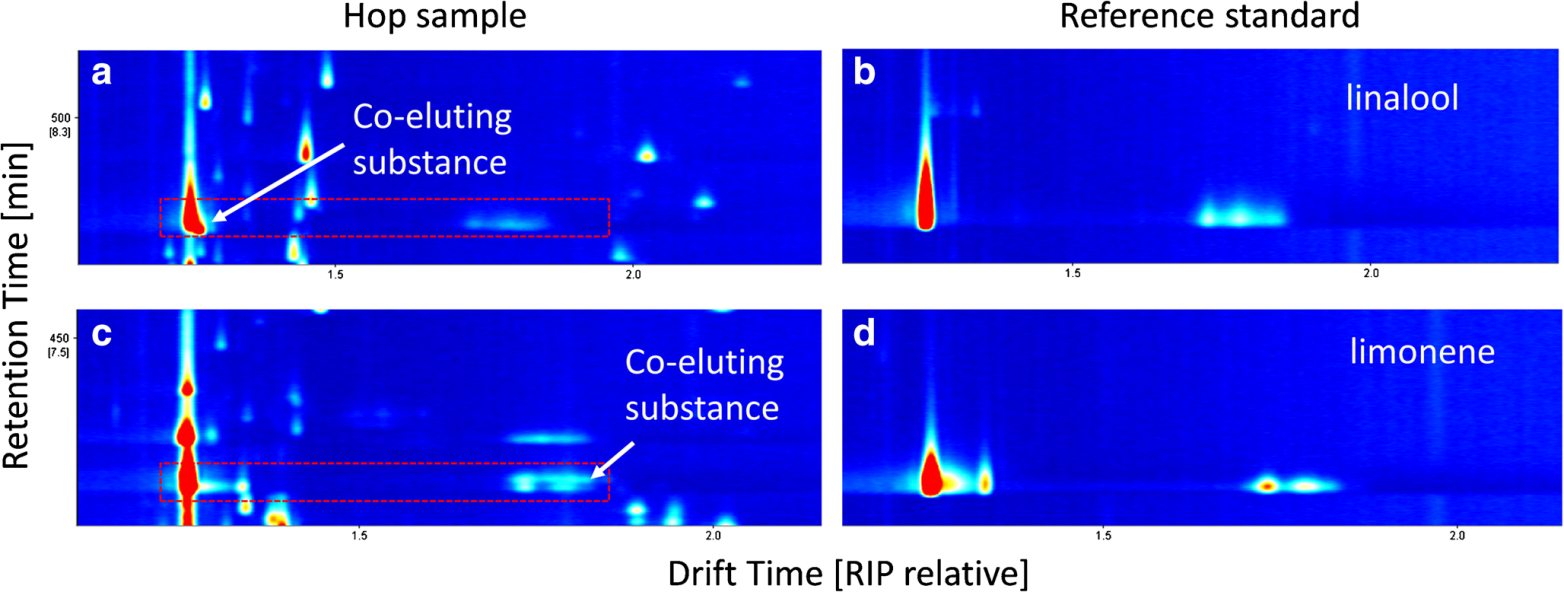
Selected areas of the IMS spectrum of the Citra hop sample (**a**, **c**) compared with reference IMS spectra of linalool (**b**) and limonene (**d**) of the 1 mg/g stock solution in sunflower oil

With regard to substance identification, the low match quality values of limonene (38) and linalool (46) either indicate a co-elution event or did not show any match. Again, IMS spectra provided much more usable information. In Fig. 2, the elution zone of linalool and limonene in a hop sample is compared with a reference standard spectrum. It can be seen that there is an overlap of an unknown ion with the monomer ion of linalool in the hop sample (Fig. 2a), when being compared with the reference spectrum (Fig. 2b). For limonene, no overlap can be seen in the monomer region, but in the dimer region (Fig. 2c), when being compared with the reference spectrum (Fig. 2d). The ion pattern and the drift times indicate tentatively another monoterpene or mono-terpenoid, however.

This example demonstrates the potential of the combination of EI-MS and IMS for differentiating substance classes in complex mixtures. With this approach, an additional dimension of information is available due to characteristic drift times, even in the case of co-elution. The drift time separation significantly increases selectivity and paired with the high sensitivity for proton affine substances such as ketones, aldehydes, and alcohols; IMS is a powerful addition to an already established EI-MS setup, if not even being a full substitution in some cases. To maximize the use of the gathered data from IMS and MS for substance identification, the *m*/*z*-based identification could be supplemented by the *K*_0_ values and the retention time indices for being used in database search, which is ongoing research at the moment. The combination of IMS and MS in the prototype used in the present study increased overall selectivity significantly and helped identifying substances, which were not identified unequivocally before.

### Aroma profiling of hop cultivars by HS-GC-IMS-MS and multivariate data analysis

The 65 different hop samples were analyzed with the prototype dual-detection setup in order to find characteristic flavor profiles and to identify cultivars with a chemically similar profile. While it is already understood that the detection of substances does not necessarily relate to their odor threshold and as such, the sensorial perception, it is still very helpful to identify similarities of hop cultivars on a molecular level.

As first step in the processing of the data, PCA was applied to both IMS and MS data for dimension reduction as well as for the visualization of possible clusters of hop samples with similar VOC profiles. As described before, the MS data featured an extremely dominant signal from the most abundant terpene myrcene, while the other compounds showed much lower intensities. This led to an overrating of the highly abundant signals, being not an optimal starting point for a PCA. For this reason, a log transformation of the MS data was performed as a pre-processing step. In contrast, IMS measurements featured an increase of higher molecular cluster ions in the case of an increasing analyte concentration. This led to a higher selectivity in the context of multivariate analysis, as the number of signals increases and not the absolute intensity of one signal only. This effect is constant under given conditions and the prerequisite that the RIP signal is still abundant.

**Fig. 3:**
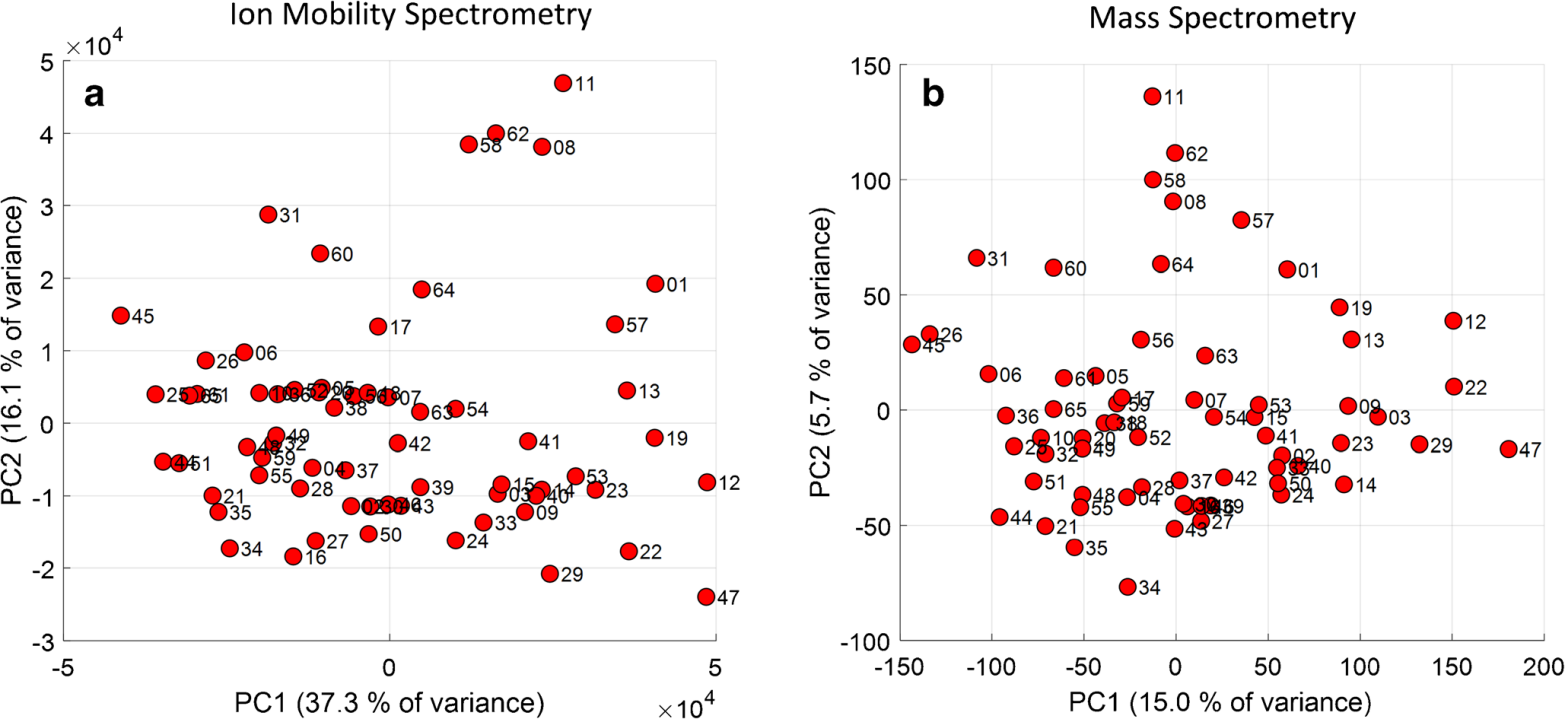
Score plot of the principal component analysis of hop aroma profiles obtained by ion mobility spectrometry (**a**) and mass spectrometry (**b**)

**Fig. 4:**
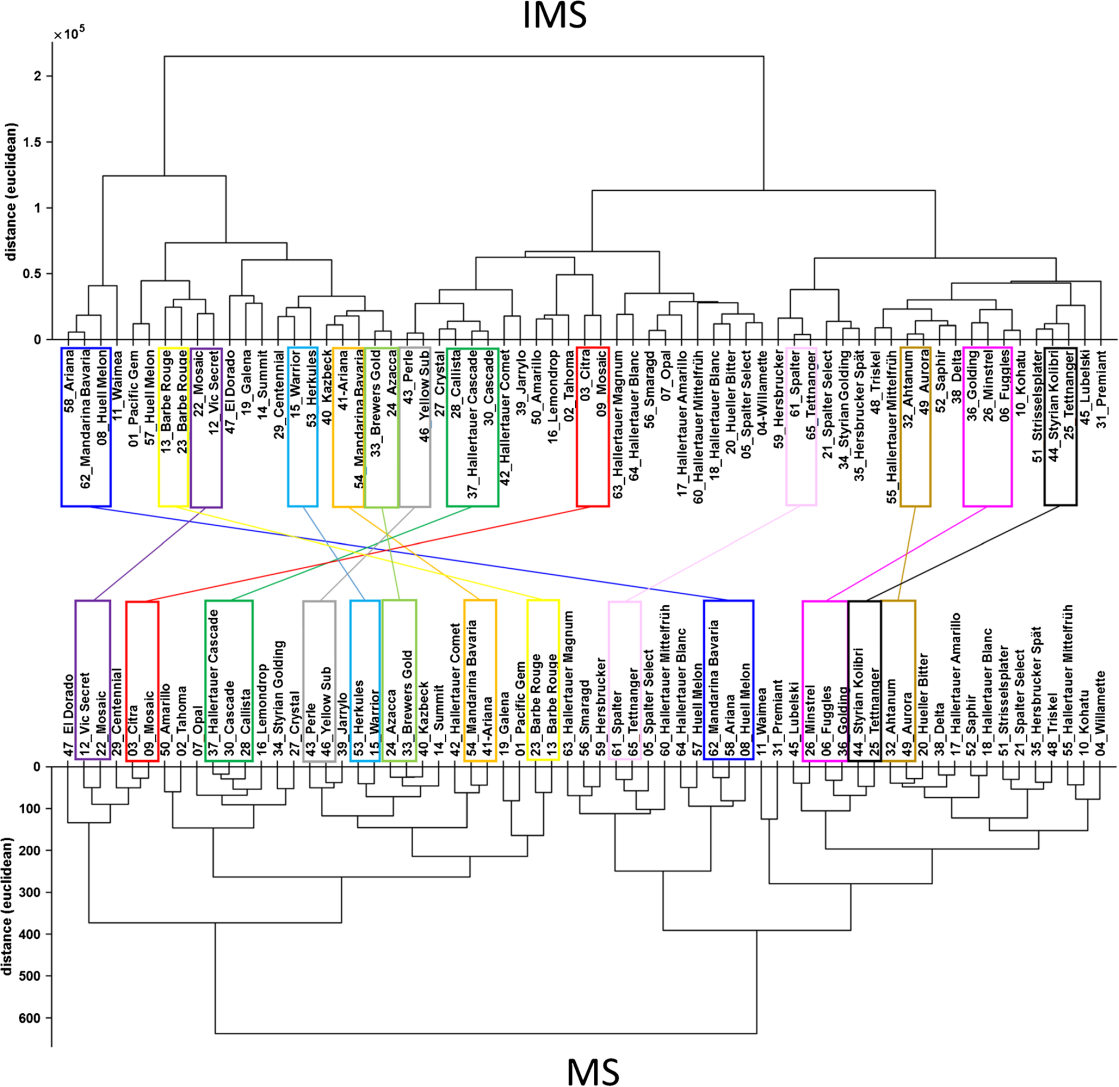
Dendrograms of the hierarchical cluster analysis obtained for the scores of the ion mobility spectrometry (top) and mass spectrometry (bottom) data with five principal components. Cultivars of interest are highlighted in colored boxes and connected by lines between both dendrograms

The results are shown in Fig. 3 as score plots of principal component (PC) 1 and 2 of the IMS and MS data, respectively. The two score plots clearly differ from each other regarding the relative location of the samples and the explained variance. The latter is an effect of the log transformation, reducing overall variance. The explained variance (EV) in MS data is only 20.7% for the first 2 PCs; the EV for the IMS data is 53.4%. In general, this can be interpreted in such way that the profiles of the hop cultivars seem to be highly different and not characterized being of high covariance. Consequently, more principal components were used and HCA was applied to the PCA data by including the first five PCs. The dendrograms obtained are plotted in Fig. 4. Small Euclidean distances indicate high similarities of the VOC profiles. Clusters of cultivars obtained in both dendrograms are highlighted in colored boxes and connected by lines.

As already expected from the score plots shown in Fig. 3, the dendrograms from both techniques differ from each other, but in some cases provide similar clusters of the hop samples investigated (highlighted cultivars in boxes). Although for both data sets the VOC profile was analyzed, the data contained different information, due to differences in selectivity and sensitivity of the detection systems. The hops included in the present sample set were obtained from different harvesting years, with different climatic conditions, harvesting dates, storage conditions, and storage periods. In summary, they differ in state of aging. Thus, it is possible that hops that differ from each other in fresh state could now show more similarities and the opposite case, hops of similar flavor in fresh state could now differ from each other. However, in both dendrograms, the special flavor hops cv. Ariana (sample no. 58), cv. Mandarina Bavaria (no. 62), and cv. Huell Melon (no. 08) from the year 2017 (dark blue box) form one cluster of small Euclidean distances, while cv. Mandarina Bavaria (no. 54) and Ariana (no. 41) from the year 2018 (orange box) form an own cluster with high Euclidean distances to the cluster from the year 2017. Cv. Huell Melon (no. 57) from the year 2016 shows smaller Euclidean distances to the cluster of the year 2017. Finally, the hop cultivars show similarities within one harvesting year and clearly differ between different harvesting years. The corresponding IMS spectra are shown in Fig. S2 in the ESM. Cultivars from the years 2018 and 2016 seem to have higher contents of certain terpenes such as limonene and α-humulene compared with the cultivars from the year 2017. This observation is in accordance with the results described by Gahr and Schüll [32], who report more intense differences of flavor compounds induced by differing climate conditions between harvesting years compared with varietal differences of the cultivars Ariana and Callista. In the present study, cv. Callista (no. 28) shows small Euclidean distances compared with the hop cultivars Cascade (no. 30), cultivated in the USA, and Hallertauer Cascade (no. 37), cultivated in Germany (corresponding IMS spectra, ESM Fig. S3). High degrees of similarity for the aroma profile of cv. Cascade and cv. Hallertauer Cascade are also reported in the literature [33].

HCA can serve as a fast and simple method for comparing VOC profiles of a high number of different hop samples. Initial indications are given about similarities that can then be characterized more detailed by investigating the raw spectra. The comparison of flavor profiles is of special concern with regard to breeding new cultivars of special flavor hops or of hops more resistant against the adverse effects of climate change, pests, and diseases [3].

### Possible correlations between the VOC profile and the α-acid content

**Fig. 5:**
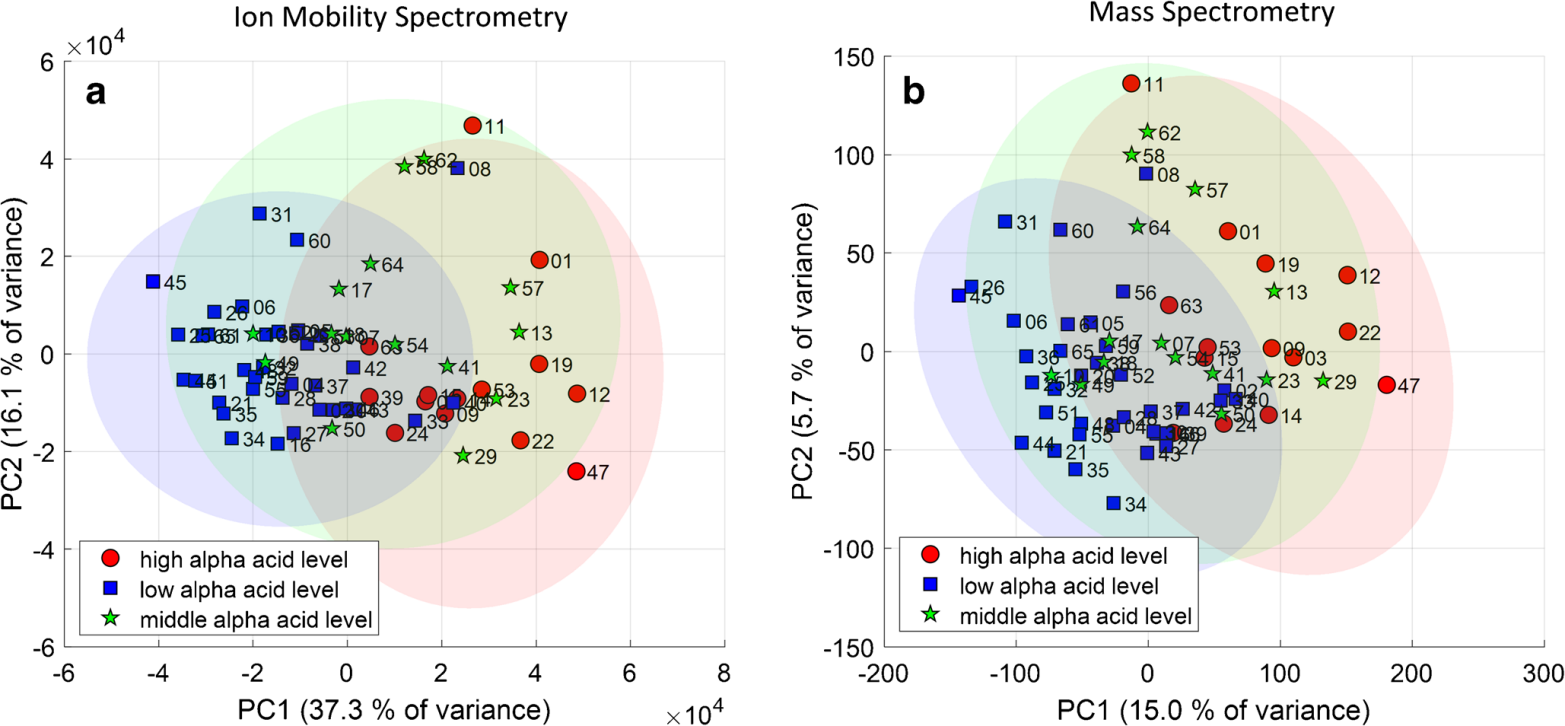
Score plot of the principal component analysis of hop aroma profiles obtained by ion mobility spectrometry (**a**) and mass spectrometry (**b**) after grouping with 97.5% confidence ellipses of the squared Mahalanobis distances

When taking a closer look to the sample distribution in the score plots (Fig. 3), it is remarkable that hop samples with very low α-acid content, such as cv. Lubelski (2%) and cv. Strisselspalter (1.8%), are located at the outer left side of PC 1, while hop samples with very high α-acid content, such as cv. Vic Secret (18.1%) or cv. Eldorado (15%), are located at the outer right side of PC 1. This observation indicated that the VOC profile of hops is affected by the α-acid content, which becomes even more obvious from the results shown in Fig. 5. Here, the hop samples were assigned to three groups: “low α-acid level hops” for α-acid levels from 1% (% w/w) to 6.9% (36 samples), “middle α-acid level hops” from 7.0 to 10% (15 samples), and all remaining samples of higher content to “high α-acid level hops” (14 samples).

**Fig. 6:**
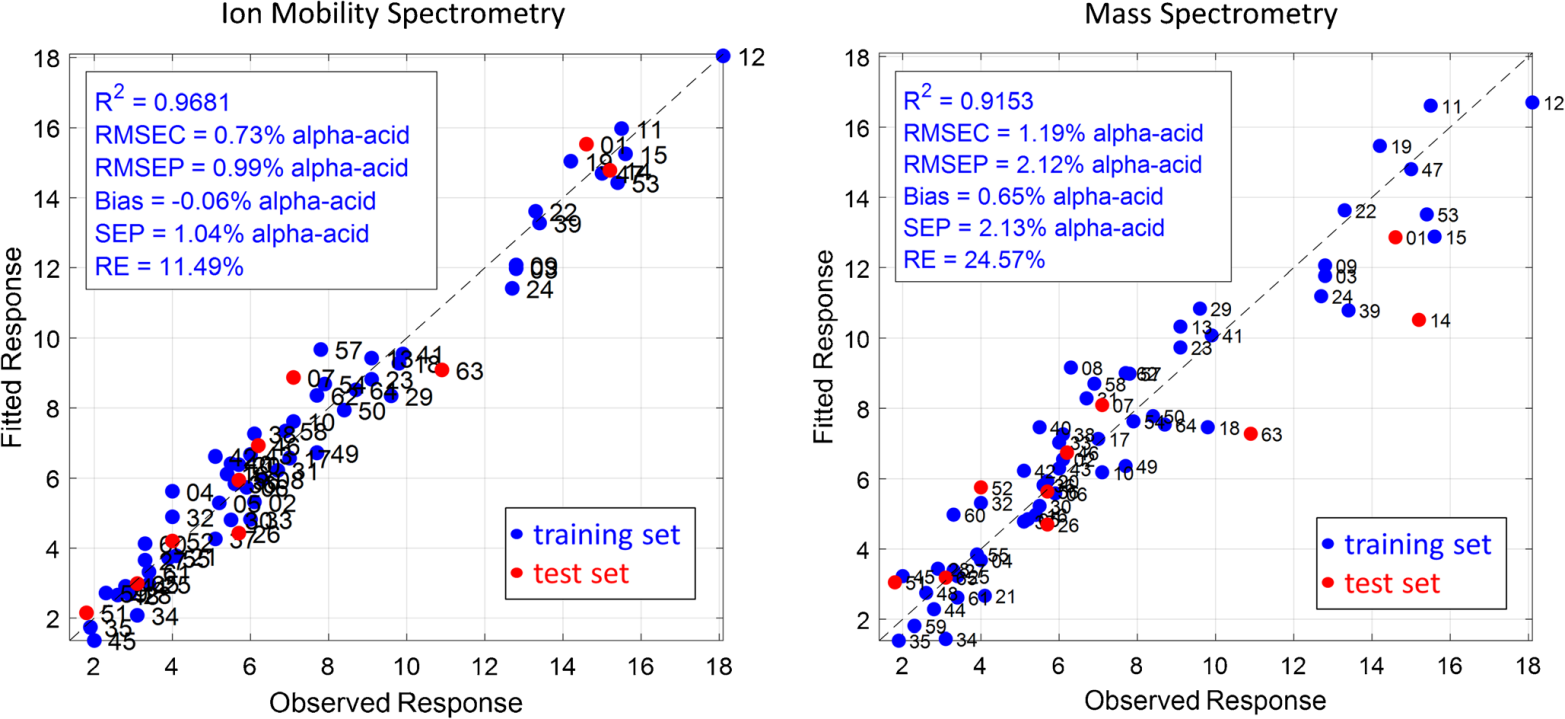
Regression plots of the partial least square regression (PLSR) of the ion mobility spectrometry data (left) and mass spectrometry (right) data correlated to the α-acid content (% w/w) of the hop samples. The figures of merit are the determination coefficient (*R*^2^), the root mean square error of correlation (RMSEC), the root mean square error of prediction (RMSEP), the systematic error (Bias), the standard error of prediction (SEP), and the relative percentage error of prediction (RE)

Although no clustering occurred, a general trend was observed. The samples seem to be separated horizontally along the first PC. For illustration, the 97.5% tolerance ellipses of the squared Mahalanobis distances are plotted. In both models, the three groups clearly overlap, and the middle α-acid level hops can be considered transitional group between the low and high α-acid level hops. Of course, the observed sample distribution could be randomly, e.g., due to cultivar differences appearing in the VOC profile. To prove this, PLSR was applied by correlating the data to the corresponding α-acid values, given from the package labelling of the hop samples. Therefore, the IMS and MS data were split into a training set (blue dots) of 55 samples and a test set (red dots) of 10 samples, distributed all over the full calibration range, respectively. Hop samples of low α-acid content clearly dominated the training set data, but they reflected the distribution of bitter and aroma hops of the actual hop variety list published by the *International Hop Grower’s Convention* (IHGC) in the year 2018 very well, where only 67 hops (25%) of 266 total hop varieties are bitter hops [34]. The regression plots and the figures of merit of the PLSR results obtained with IMS and MS data are shown in Fig. 6.

Pareto scaling was applied to the IMS data as additional pre-processing step from which an improvement of the RE from 13.51% without scaling to 11.49% with scaling could be obtained. No improvement was observed for the RE of the MS data. Here, a decrease from 24.57% without scaling to 27.64% with scaling was obtained. Generally, in PLSR, the calibration can be improved continuously by increasing the number of LV for model building. However, there is a high risk of overfitting. It must be kept in mind that the information content in the data and, therefore, in the LVs is limited. The higher the number of chosen LVs, the higher the risk of including random noise. An indication of overfitting is an increasing calibration error, while the prediction error decreases. Considering that the best model fit was achieved for the IMS data with five LVs and for the MS data with two LVs (Fig. 6). For both techniques, a clear correlation can be observed between the VOC profiles and the corresponding α-acid level of the hop samples, with a *R*^2^ of 0.9681 and a RMSEC of 0.73% α-acid for the IMS data and with a *R*^2^ of 0.9153 and a RMSEC of 1.19% α-acid for the MS data. However, for the prediction of the α-acid level calculated with an external test set, the model built with IMS data shows much smaller error rates than the model built with MS data. The RE is 11.49% for the IMS data, with a RMSEP of 0.99% α-acid and a SEP of 1.04% α-acid. For MS data, the RE of 24.57%, the RMSEP of 2.12% α-acid, and the SEP of 2.13% α-acid of the MS data lie clearly above. These results demonstrate the high importance of an independent test set. While the PLSR calibration results of the MS data appear promising, the prediction results show a comparatively poor model fit. Killeen et al. applied PLSR on spectroscopic data measured by IR, NIR, and Raman correlated to HPLC quantification results and obtained RMSEC and RMSEP values between only 1.2 and 1.8% α-acid for all three techniques [19]. The error values in the present study, obtained by applying PLSR on IMS spectra, are comparable and even slightly lower. It should be noted that the actual α-acid content of the hop samples was not analyzed, but only taken from the manufacturers’ specifications. The α-acids are reported to be relatively stable under inert storage conditions at low temperatures [1, 11, 12]. Nonetheless, the models could be even better, when the actual values would have been considered. However, the results give raise to the question, why the volatile profile can be correlated to the non-volatile α-acids and why the IMS data are more suitable for the regression model than the MS data. One possibility could be that degradation and reaction products of the α-acids are detectable in the volatile profile. Kishimoto et al. identified some esters of increased concentrations in beer hopped with aged hops and proposed that these components were formed by esterification of short-chain fatty acids derived from the degradation of α-acids [35]. Thus, high α-acid hops could contain such degradation products in higher amounts than low α-acid hops. In addition, Sharpe and Laws report a correlation between the amounts of α-acids and myrcene [13]. It was shown by Hartley that the loss of α-acids during storage was higher in hops with higher contents of essential oils and he postulated that myrcene could initiate the oxidation of α-acids by the formation of peroxides [13, 36]. Another possibility could be that low α-acid level hops simply contain higher levels of essential oils, because they are often used as aroma hops. In contrast, Aberl and Coelhan [9] showed that bitter hops, providing high α-acid level hops, tend to contain higher levels of essential oils compared with aroma hops. In addition, Patzak et al. describe a positive correlation of the number and size of lupulin glands with the content of bitter acids in different hop cultivars [37]. In this context, it is important to note that α-acids and terpenes are synthetized via the same biosynthetic pathway and their composition is characteristic for the genotype of a hop cultivar [38, 39]. While bitter acids are located exclusively in the glandular trichomes of the hop cones, terpenes are mainly present in the trichomes, and also accumulate in further plant compartments [38]. More precisely, mono- and sesquiterpenes are formed from the two basic precursors dimethylallyl pyrophosphate (DMAPP) and isopentenyl diphosphate (IPP), while bitter acids are formed by prenylation of polyketides by DMAPP [38–40]. IPP that can be converted to DMAPP by IPP isomerase is produced in the cytosolic mevalonate (MVA) pathway and the plastidial deoxyxylulose phosphate (MEP) pathway, the so-called non-mevalonate pathway. Goese et al. show with isotope labelling that the bitter acids are primarily deriving from the MEP pathway [39], as well as monoterpenes, while sesquiterpenes can be more assigned to the MVA pathway in plants in general, but not exclusively [39, 40]. However, Nagel et al. found out by enzyme analysis of hop glandular trichomes that the MEP predominates as building pathway for the essential oils in hops [41]. For this reason, there is a further possibly consisting in a functional relation between the bitter acid formation and the terpene metabolism in hop trichomes.

**Fig. 7:**
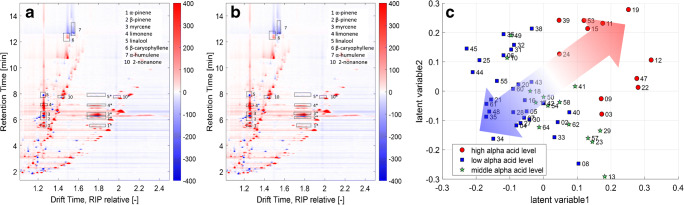
Loading plots derived from the first (**a**) and second (**b**) latent variable and score plot (**c**) of the partial least square regression of the ion mobility spectrometry data. Signals belong to α-pinene (1), β-pinene (2), myrcene (3), limonene, linalool (5), β-caryophyllene (6), α-humulene (7), and 2-nonanone (10)

To investigate which substances are mainly responsible for the observed correlation, the loading and score plots of the first two LVs of the IMS data can be considered (Fig. 7).

**Fig. 8:**
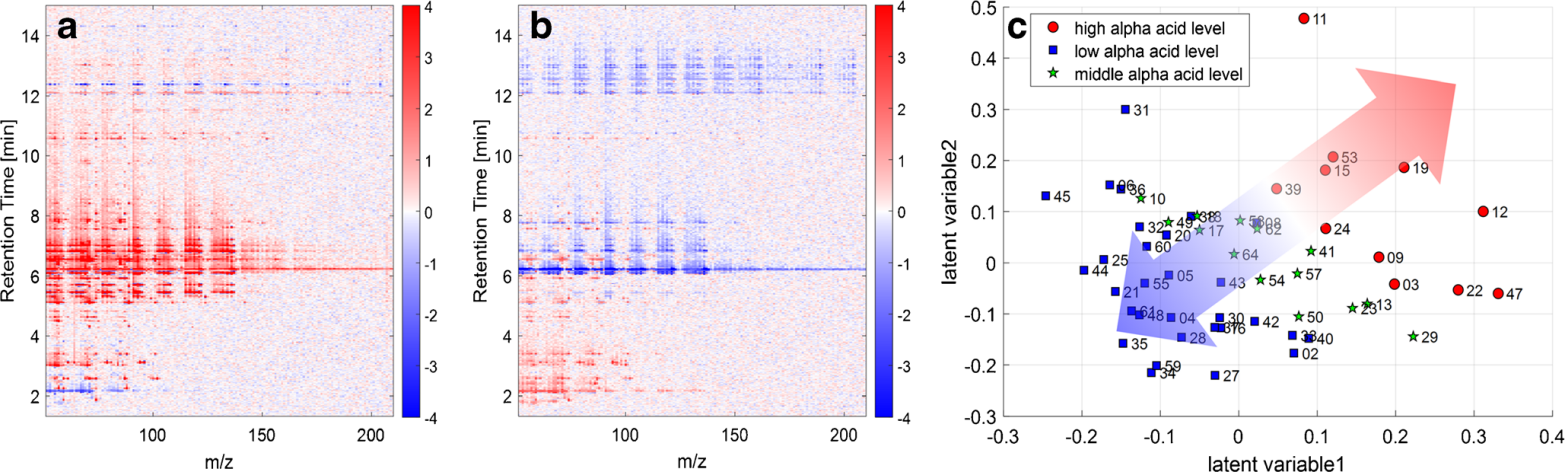
Loading plots derived from the first (**a**) and second (**b**) latent variable and score plot (**c**) of the partial least square regression of the mass spectrometry data

However, not one single specific substance only shows very high (red) or low (blue) loading values (Fig. 7a and b). There are signals in all regions of the spectrum from all substance classes that show intensive loading values. This fact indicates that not one single compound is responsible for the correlation, but rather the concentration ratio of several compounds. In consideration of the corresponding score plot (Fig. 7c), samples of positive score values in LV1, in particular high α-acid level hops, contain higher amounts of α-pinene (1), β-pinene (2), myrcene (3), limonene (4), and β-caryophyllene (6) and lower levels of linalool (5) and α-humulene (7) compared with samples of negative score values in LV1. For low α-acid hops, this ratio is inverse. The arrow in Fig. 7c with corresponding color gradient illustrates the separation. Also, minor volatile compounds that do not belong to the terpenes seem to have an impact on the correlation and show high and low loading values. Samples of negative score values in LV2 contain higher amounts of terpenes and lower amounts of minor volatile compounds compared with samples with positive loading values. Additionally, the signals of certain monoterpenes are less intensive in LV2 compared with LV1 and seem to have less impact on LV2. Attention should be paid to compounds of similar RTs. They are resolved in IMS, but cannot be detected or differentiated in MS. For comparison, the loading and score plots of the two first LVs obtained with the MS data are shown in Fig. 8. Subsequently, this information is not accessible from the MS data. That could be one reason for the higher errors of prediction for the MS data obtained in Fig. 6.

The results indicate a relationship between the metabolism of volatile aroma compounds in hops and the biosynthesis of α-acids. To investigate the origin of the observed correlation, an extensive study with fresh hops directly from pelletization should be done to exclude the influence factor of storage. At the current state, it seems plausible that certain terpenes mostly or exclusively formed in the lupulin trichomes dominate in hops rich in lupulin glands, while there is also a positive correlation postulated between the degradation of α-acids and essential oils [36].

## Conclusion

The prototype HS-GC-MS-IMS setup presented herein allows the simultaneous generation of complementary IMS and MS data. With a soft ionization and drift time-based ion separation on the one hand and a hard ionization and *m/z*-based separation on the other hand, substance identification in the case of co-elution is improved, substantially. An additional make-up gas flow after the column split reduces peak broadening and high deviations in RT between the both detection systems. In this study, HS-GC-MS-IMS was used for the analysis of the highly complex VOC profile of hop (*Humulus lupulus* L.) as example for application. Growing conditions, harvesting time, and processing and storage conditions influence the hop quality. Routinely applied analytical methods for hop quality assurance are either time-consuming and cost-intensive or less meaningful. The usage of HS-GC-MS-IMS in combination with chemometrics such as PCA and HCA enables a fast search for similarity between the flavor profiles of different hop cultivars. When using HCA, initial indications are given for similarities in the aroma profiles of certain hop cultivars within the same harvesting year or cultivated in different growing areas. Furthermore, the results of the PCA indicate a relationship between the α-acid levels and the aroma profile of hops. PLSR was applied to the IMS data leading to a RMSEC of only 0.73% α-acid next to a RMSEP of 0.99% α-acid and a SEP of only 1.04% α-acid. Further investigations are needed to understand this relationship, but the loading and score plots of the PLSR model indicate that the biosynthesis or degradation of α-acids and terpenes results in a specific ratio of some aroma compounds in the VOC profile of hops. With regard to degradation processes taking place during storage, a more extensive study with fresh hop pellets is required.

In conclusion, HS-GC-MS-IMS or even HS-GC-IMS on its own in combination with intelligent machine learning strategies is a promising tool for the future quality control of brewing hops; however, further research will be necessary. In particular, this approach could help to objectify sensory assessments and to identify differences or similarities between hop cultivars from different harvesting years or growing regions. Compared with the standard EBC methods, no time-consuming extraction procedures or hazardous chemicals, such as lead acetate or diethyl ether, are required. The quality status of hop including the volatile aroma profile and possibly the α-acid level can be recorded by one single injection only.

## Electronic supplementary material

ESM 1 (PDF 776 kb)
