# Supplementary material for: Gas-phase volatilomic approaches for quality control of brewing hops based on simultaneous GC-MS-IMS and machine learning
Source: Anal Bioanal Chem. 2020 Aug 4;412(26):7085–97. doi: 10.1007/s00216-020-02842-y (PMC7497504; doi:10.1007/s00216-020-02842-y)
Supplement: Supplementary file 1 — (PDF 776 kb) [file 216_2020_2842_MOESM1_ESM.pdf]

Analytical and Bioanalytical Chemistry

Electronic Supplementary Material

Gas-phase volatilomic approaches for quality control of brewing hops based on simultaneous GC-MS-IMS and machine learning

Rebecca Brendel, Sebastian Schwolow, Sascha Rohn, Philipp Weller

Table S1 List of hop samples

Number	Name	Origin	α-acid [%w/w]	Harvest year
1	Pacific Gem	NZ	14,6	2017
2	Tahoma	USA	6,1	2017
3	Citra	USA	12,8	2017
4	Willamette	USA	4	2017
5	Spalter Select	DE	5,2	2017
6	Fuggles	GB	5,9	2017
7	Opal	DE	7,1	2017
8	Huell Melon	DE	6,3	2017
9	Mosaic	USA	12,8	2017
10	Kohatu	NZ	7,1	2017
11	Waimea	NZ	15,5	2017
12	Vic Secret	AUS	18,1	2018
13	Barbe Rouge	FR	9,1	2017
14	Summit	USA	15,2	2016
15	Warrior	USA	15,6	2017
16	Lemondrop	USA	5,4	2017
17	Hallertauer Amarillo	DE	7	2017
18	Hallertauer Blanc	DE	9,8	2018
19	Galena	USA	14,20	2016
20	Hueller Bitter	DE	5,7	2015
21	Spalter Select	DE	4,1	2018
22	Mosaic	USA	13,3	2018
23	Barbe Rouge	FR	9,1	2017
24	Azacca	USA	12,7	2017
25	Tettnanger	DE	3,4	2018
26	Minstrel	GB	5,7	2018
27	Crystal	USA	3,3	2018
28	Callista	DE	2,9	2018
29	Centennial	USA	9,6	2017
30	Cascade	USA	5,5	2018
31	Premiant	CZ	6,7	2017
32	Ahtanum	USA	4	2017
33	Brewers Gold	DE	6	2018
34	Styrian Golding (Celeia)	SLO	3,1	2018
35	Hersbrucker Spät	DE	1,9	2018
36	Golding	GB	5,6	2017
37	Hallertauer Cascade	DE	5,1	2018
38	Delta	USA	6,1	2017
39	Jarrylo	USA	13,4	2017
40	Kazbek	CZ	5,5	2018
41	Ariana	DE	9,9	2018
42	Hallertauer Comet	DE	5,1	2018
43	Perle	DE	6	2018
44	Styrian Kolibri	SLO	2,8	2018
45	Lubelski	PL	2	2018
46	Yellow Sub	DE	6,2	2018
47	El Dorado	USA	15	2017
48	Triskel	FR	2,6	2018
49	Aurora	SLO	7,7	2018
50	Amarillo	USA	8,4	2017
51	Strisselspalter	FR	1,8	2018
52	Saphir	DE	4	2017
53	Herkules	DE	15,4	2018
54	Mandarina Bavaria	DE	7,9	2018
55	Hallertauer Mittelfrüh	DE	3,9	2018
56	Smaragd	DE	5,7	2017
57	Hüll Melon	DE	7,8	2016
58	Ariana	DE	6,9	2017
59	Hersbrucker	DE	2,3	2016
60	Hallertauer Mittelfrüh	DE	3,3	2017
61	Spalter	DE	3,4	2017
62	Mandarina Bavaria	DE	7,7	2017
63	Hallertauer Magnum	DE	10,9	2018
64	Hallertauer Blanc	DE	8,7	2018
65	Tettnanger	DE	3,1	2018

AUS= Australia, CZ= Czech Republic, DE= Germany, FR= France, GB= Great Britain, NZ= New Zealand, PL= Poland, SLO= Slovenia, USA= United States of America

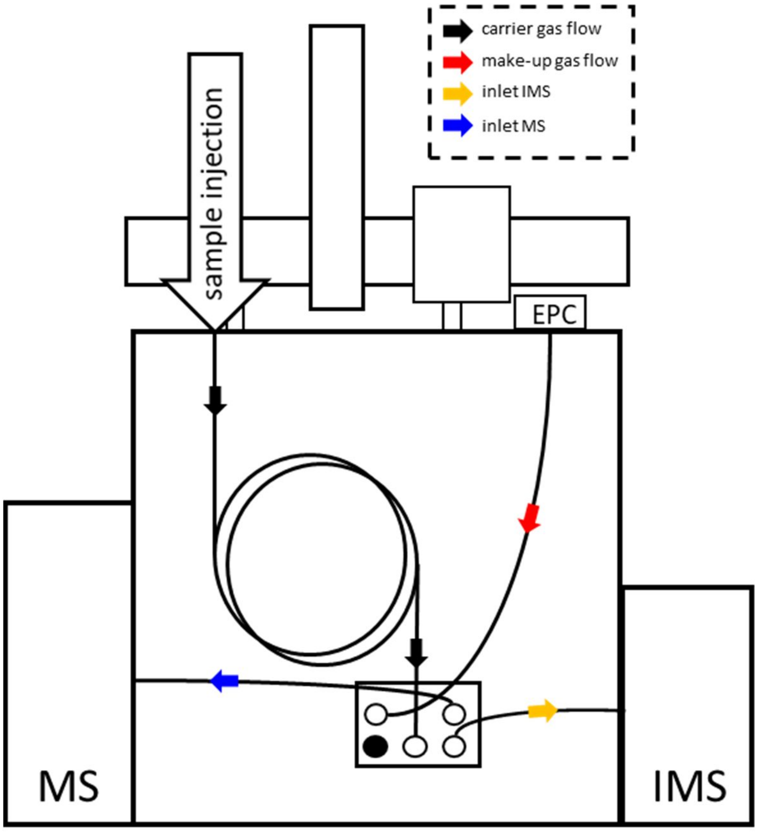

Fig. S1 Setup of the HS-GC-MS-IMS system with electronic pressure control (EPC)

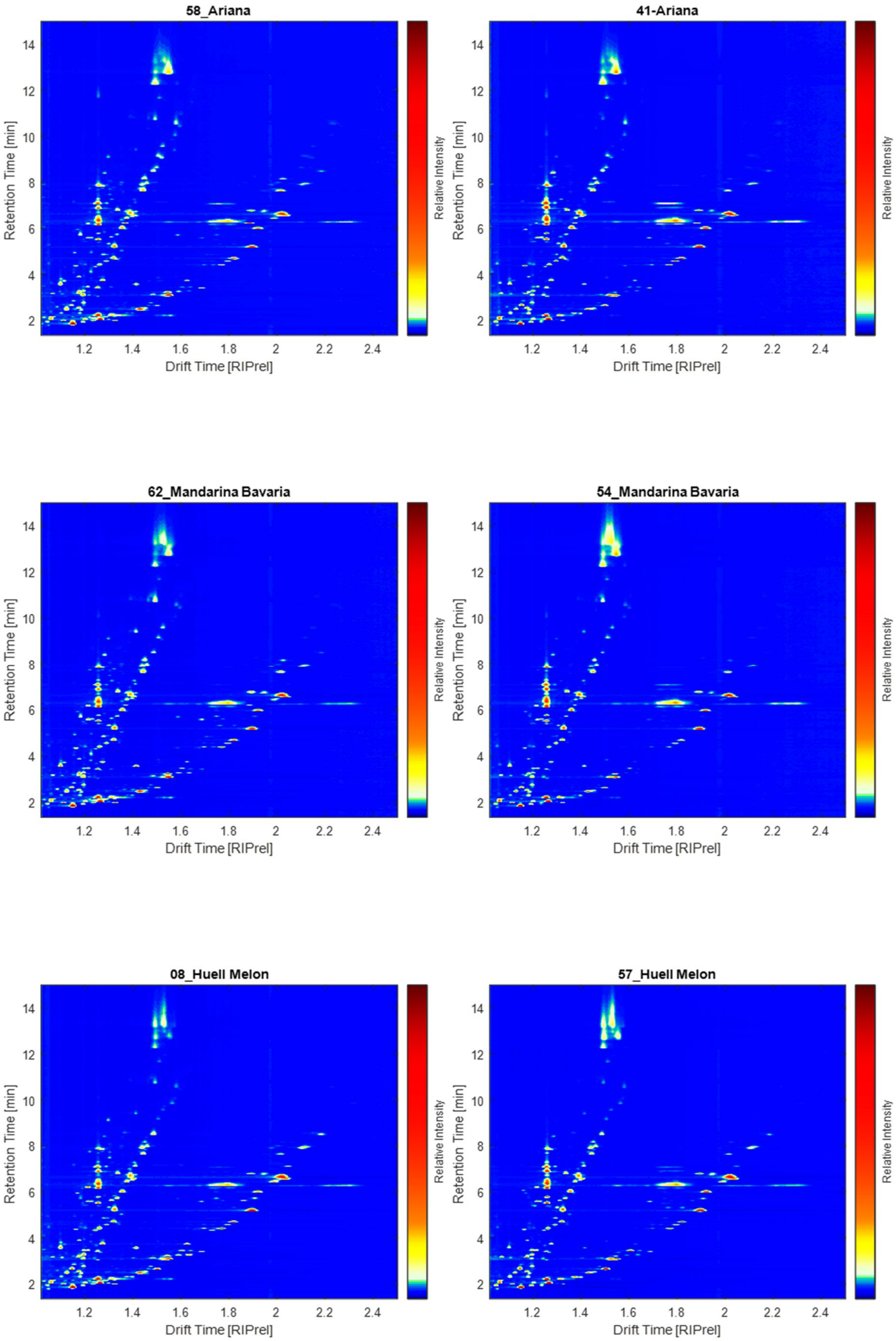

Fig. S2 The ion mobility spectra of the hop cultivars Ariana, Mandarina Bavaria and Huell Melon from different harvest years

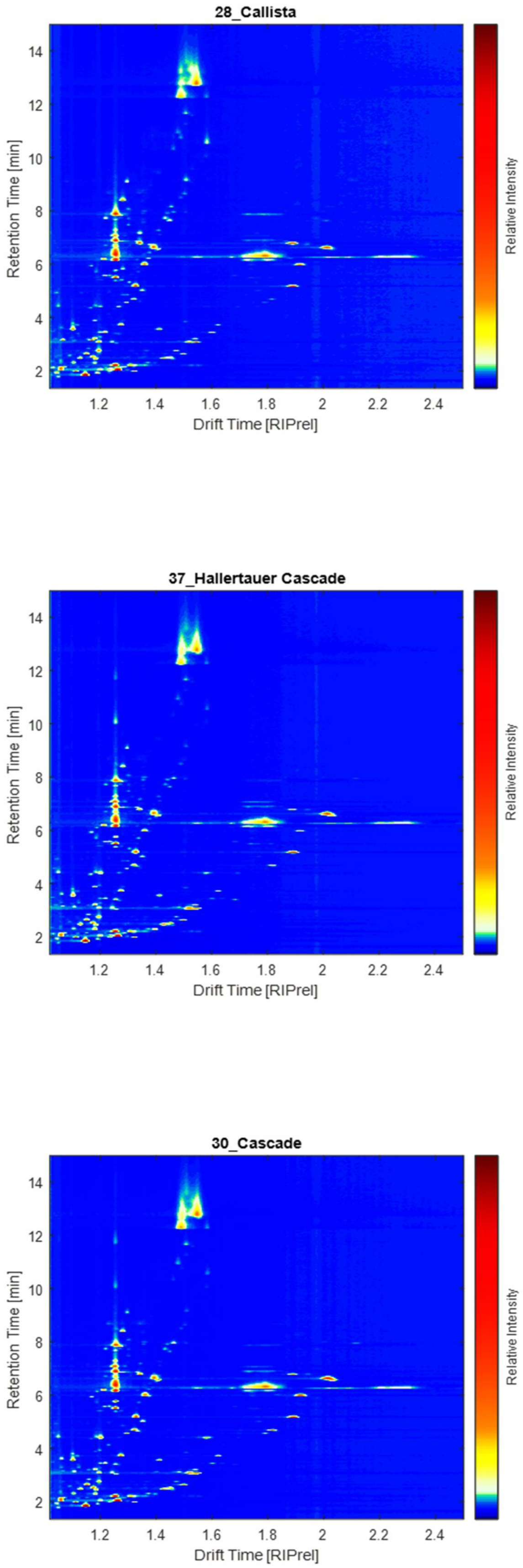

Fig. S3 The ion mobility spectra of the hop cultivars Callista, Hallertauer Cascade and Cascade
